# The study of the roundness and cylindricity deviations of parts produced with the use of the additive manufacturing

**DOI:** 10.1007/s00170-022-09838-1

**Published:** 2022-07-29

**Authors:** Jacek Świderski, Włodzimierz Makieła, Tomasz Dobrowolski, Krzysztof Stępień, Uros Zuperl

**Affiliations:** 1 Faculty of Mechatronics and Mechanical Engineering, Kielce University of Technology, Al. 1000-lecia P. P. 7, PL-25314 Kielce, Poland; 2 Faculty of Mechanical Engineering, University of Maribor, Smetanova ulica 17, SI-2000 Maribor, Slovenia

**Keywords:** Additive manufacturing (AM) technology, Surface texture, Geometrical product specification (GPS), Roundness, Cylindricity

## Abstract

The paper is dedicated to the evaluation of the accuracy of rotary parts produced with the use of advanced manufacturing technology. The authors investigated the impact of the layer thickness of the applied material and the orientation of the model when printing using the PolyJet method™ on the geometrical quality of manufactured products. To analyze the influence of the assumed factors on the geometrical quality of the holes, a novel evaluation method has been developed. The proposed method takes into account parameters such as roundness deviation, profile irregularity coefficient, dominant harmonic component of the roundness profile, cylindricity deviation, diameter error, and surface topography parameters. The study presented in this paper had two main objectives. The former was to analyze the impact of the layer thickness of the applied material and the orientation of the model when printing using the PolyJet method™ on the geometrical quality of rotary parts. The latter objective was to test a novel, multi-parametric method of evaluation of the accuracy of produced parts in practice. The results obtained by the authors prove that the new evaluation method can be useful in the assessment of the accuracy of manufactured products.

## Introduction

Growing competition in a global market is the reason why contemporary industry requires devices that will reduce production costs and time and enable the manufacturing of short series or single objects without the need to involve large financial means. These requirements are met by the so-called additive manufacturing [1–4].

Additive manufacturing technologies have been known for over 30 years. According to ISO standard (ISO 52900), additive fabrication is defined as follows [1, 4]: “Manufacturing processes which employ an additive technique whereby successive layers or units are built up to form a model.”

Additive manufacturing technologies find numerous practical applications in the aerospace industry [5–7], automotive, biomedicine [7], dentistry [8], power industry, and in other fields.

The possibility of relatively low-cost production of short series or single copies of geometrically complex objects makes the medical industry one of the main areas of application of additive technologies [5]. Additive manufacturing technologies are used, for example, to fabricate implants, prostheses, or tissues that can be inserted into the human body [1]. An important field of the application of additive manufacturing is producing magnetoactive soft materials, which is described in [9]. It should be noted that additive manufacturing can be useful during emergency situations such as COVID-19 [10].

Additive manufacturing can be classified as follows: photopolymerization, materials extrusion, sheet lamination, powder bed fusion, binder jetting, materials jetting, and directed energy deposition. 3D printing technology is capable of producing fully functional parts from a wide range of materials, including ceramics, metals, polymers, and their combinations in the form of hybrid and composite materials. Currently, there are more than 350 industrial AM machines on the market, which use more than 450 various materials [1, 2].

The discussion about which technology performs better than the others is useless, as each of them has its own target applications. Nowadays, 3D printing technologies are no longer limited to manufacture prototypes, but are increasingly being used to produce a variety of end products as well.

PolyJet technology is based on printing elements from liquid photopolymer resins cured with UV light. As with traditional inkjet printers, piezoelectric print heads spray layers of liquid light-curing photopolymer onto the work table. The polymer is then cured with the use of ultraviolet (UV) light. This process, also used in stereolithography technology (SLA), is called photopolymerization.

Parts printed with the use of PolyJet technology are characterized by high accuracy while obtaining high surface smoothness. An additional advantage is the ability to build models from a wide range of materials, including hard (similar to ABS), elastic (rubber-like), and transparent.

The 3D printers offered by manufacturers can work in three modes:

- HS (high speed), characterized by the high speed of printing of the parts,+
- HQ (high quality), which permits printing elements with a lower speed but with very high accuracy, owing to the use of a layer height of 16 microns, and
- DM (digital material), which allows the combination of various types of materials.

With a layer resolution of the applied material in the range from 16 to 30 microns, thin walls and geometrically complex elements can be produced using a large number of materials with various properties.

One of the most important factors that decide about the usefulness of the manufactured parts is to give them the correct dimensions in the manufacturing process and to ensure the assumed geometrical quality of the surface of the end product will be achieved.

The research carried out so far mainly focuses on the assessment of the parameters of the surface texture of parts manufactured with the use of various printing methods.

Kumar and Kumar [11] present the results of research on the influence of surface orientation and layer thickness on the surface roughness of parts produced by the PolyJet method. The orientation of the surface was changed in the range of 0–360° with the angular step 3°. The study revealed that surface orientation is the main factor affecting surface roughness.

Beltran et al. [12] study the influence of several factors on the quality of the features of cylindrical elements of the parts manufactured by the PolyJet method. The results lead to the conclusion that this quality is mainly influenced by the orientation and size of the part, while the position of the part in the working space of the printer has a relatively smaller impact on the geometrical accuracy of the end product.

Zmarzly et al. [13] present, among others, the results of measurements of the dimensions and topography of the surface of a casting mold produced by the PolyJet Matrix (PJM) technology. It was found that the relative error of the linear dimensions of the printed element in relation to the nominal dimensions was about 1%. Surface topography analysis was carried out, taking into account the components of waviness and surface roughness.

An important problem when using additive technologies is the optimization of energy consumption. Sanders et al. [14] present an experimental study of the energy consumption during PolyJet 3D printing depending on the location of the samples on the working platform and the volume of the printed elements.

Lord Kelvin's words, “If you cannot measure it, you cannot improve it,” well reflect why measurement is an essential part of the technological process. The biggest advantage of additive manufacturing is the ability to produce almost any required shape. Immense freedom in the design of products in relation to the geometry of both external and internal surfaces requires the use of appropriate measurement methods to determine the compliance of the manufactured parts with the requirements [15].

Due to the complexity of the geometry, the characteristics of the surface layer, and the wide range of materials used in additive manufacturing, it is essential to choose the right methods to measure dimensions, geometric tolerances, and surface topography [16–18]. Contact measurements of surface topography may not be applicable in many cases due to the risk of scratching the surface by the mapping blade or the influence of temperature changes during long-term measurements [19, 20].

One of the most significant works in the area of surface topography of elements produced by additive technologies [21] indicates that such surfaces can be measured by contact and noncontact methods. However, the work [21] does not provide any information about the measurements of the form deviations of parts produced by additive manufacturing.

Similarly, the review on problems of in situ process monitoring and in-situ measurements for metal additive manufacturing presented in [22] does not provide any information on form deviations, either.

Zhang et al. [23] describe a fringe projection system for in situ metrology of laser powder bed fusion processes. The system is dedicated to measurements of surface roughness.

The authors of the work [24] demonstrate a system that allows the detection of defects of parts produced by additive manufacturing with the use of inline coherent imaging.

Tian et al. [25] study the problem of the influence of selected parameters of selective laser melting on the surface roughness of manufactured parts. To investigate this problem, the contact measurement method was applied.

Zmarzly et al. [26] focus on the surface assessment of the quality of products manufactured with the use of fused deposition modeling. Contrary to works [21–25], the authors of work [26] analyze the effect of the orientation of the part in fused deposition modeling on the value of the form deviation. In this study, the value of total roundness deviation RONt is observed.

The analysis of the literature shows that sources of knowledge about the influence of additive manufacturing process parameters on the value of form deviations are very limited. Most works concern the analysis of surface roughness. Among the scientific articles analyzed by the author, only one concerns the analysis of the impact of additive manufacturing parameters on the value of form deviations (paper [26]).

As mentioned previously, this work concerns fused deposition modeling and the impact of its parameters on the value of the roundness deviation. Therefore, the authors have made efforts to conduct more thorough research in this area. One of the cases studied was the PJM method and the influence of model orientation and layer thickness on the accuracy of manufactured holes.

In order to comprehensively assess form deviations of the manufactured parts, the authors have developed a novel method called a multi-parametric assessment method. Testing this method in practice was one of the main aims of the presented study.

## Materials and methods

Growing competition in a global market is the reason why contemporary industry requires devices that will reduce production costs and time and enable the manufacturing of short series or single objects without the need to involve large financial means. These requirements are met by the so-called additive manufacturing [1–4].

The shape of the sample was designed using SolidWorks software. The project was saved in an STL file in the form of a triangle mesh. The approximation parameters were selected in a way ensuring that the accuracy of the STL model is higher than the accuracy of the 3D printer used. A file recording mode with a linear tolerance of 0.01 mm and an angular deviation of 5° was used. The mesh of the model consisted of about 33,000 triangles.

**Fig. 1:**
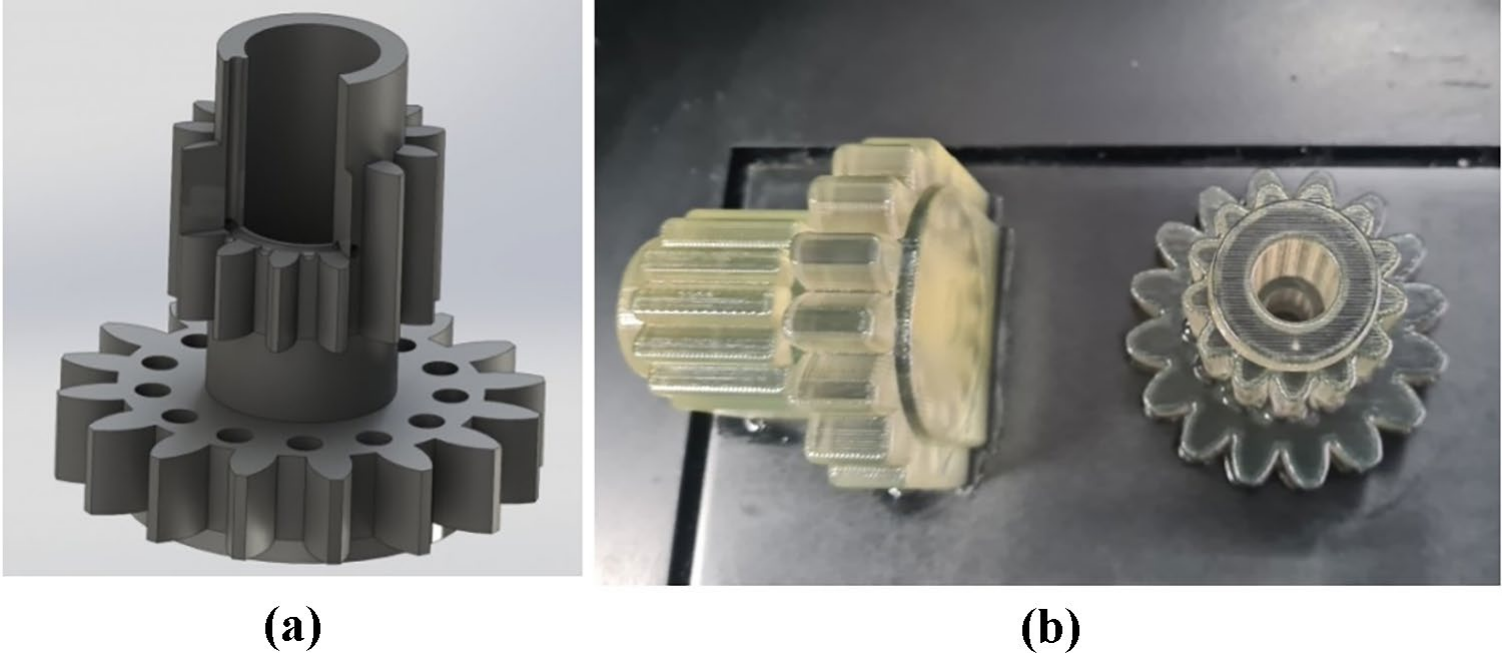
The model to be investigated: **a** CAD model of the sample. **b**) Orientation of samples on the building platform

The model has been designed in a way permitting the analysis of the accuracy of manufacturing in the process of 3D printing using the PolyJet method™ in terms of dimensional accuracy, geometric tolerances such as tolerances of form, direction and position, and surface texture quality (Fig. 1).

The models were arranged on the printer’s working platform in two distinctive orientation variants, as shown in Fig. 1.

The samples were produced using a Connex 350TM 3D printer applying the PolyJet™ method. The printer allows printing models in a wide range of colors. It allows the use of three resins simultaneously in one printing process without the need to change the material. Mixing STRATASYS resin permits obtaining 82 building materials. In high-quality mode, the thickness of the applied resin layer is 16 µm, and in high-speed mode, it is 30 µm. The manufacturing accuracy declared by the producer is from 20 to 85 microns for models with dimensions below 50 mm and 200 microns for larger models.

For the manufacturing of sample models, MED610 material was used. MED610 is a transparent, biocompatible PolyJet material™ medically approved for contact with the human body. Med610 is a photo polymer resin, and its mechanical properties are given in Table 1.

**Table 1:** Mechanical properties of the material MED610

**Properties**	**Value**
Tensile strength	50–65 MPa
Modulus of elasticity	2000–3000 MPa
Hardness (Rockwell scale)	73–76 HRM
Elongation at break	10–25%

The thickness of the layer (the height of the layer) was 16 μm and 30 μm. The printed parts have been denoted in accordance with Table 2.

**Table 2:** Notations of manufactured parts

**Notations**	**Layer height, µm**	**Orientation**
16V	16	Vertical
16H	16	Horizontal
30V	30	Vertical
30H	30	Horizontal

To assess the geometrical accuracy of the manufacturing process, the parts were measured with the use of the Prismo Navigator coordinate measuring machine with a Vast Gold central scanning head with a maximum permissible error MPE = 0.9 + L/350 μm (where L is the measured length, mm) and with the use of the system for roundness and cylindricity measurements Talyrond 365 by Taylor Hobson.

The surface roughness parameters of the parts were measured by the scanning coherence interferometer Talysurf CCI [16].

Measurements and determination of the parameters of the surface texture, as well as parameters of roundness and cylindricity, were carried out in accordance with relevant standards [27–32].

## Results and discussion

In order to carry out this research, four parts were produced with the use of PJM technology. The first couple of parts was fabricated at a horizontal position, while the second was fabricated at a vertical one. Each couple of parts in a given orientation was manufactured with the use of different layer thicknesses. The applied layer thickness applied was equal to 16 microns and 30 microns, respectively. All measurements, whose results are given in this work, were repeated 3 times, and the average value was taken for the analysis.

**Fig. 2:**
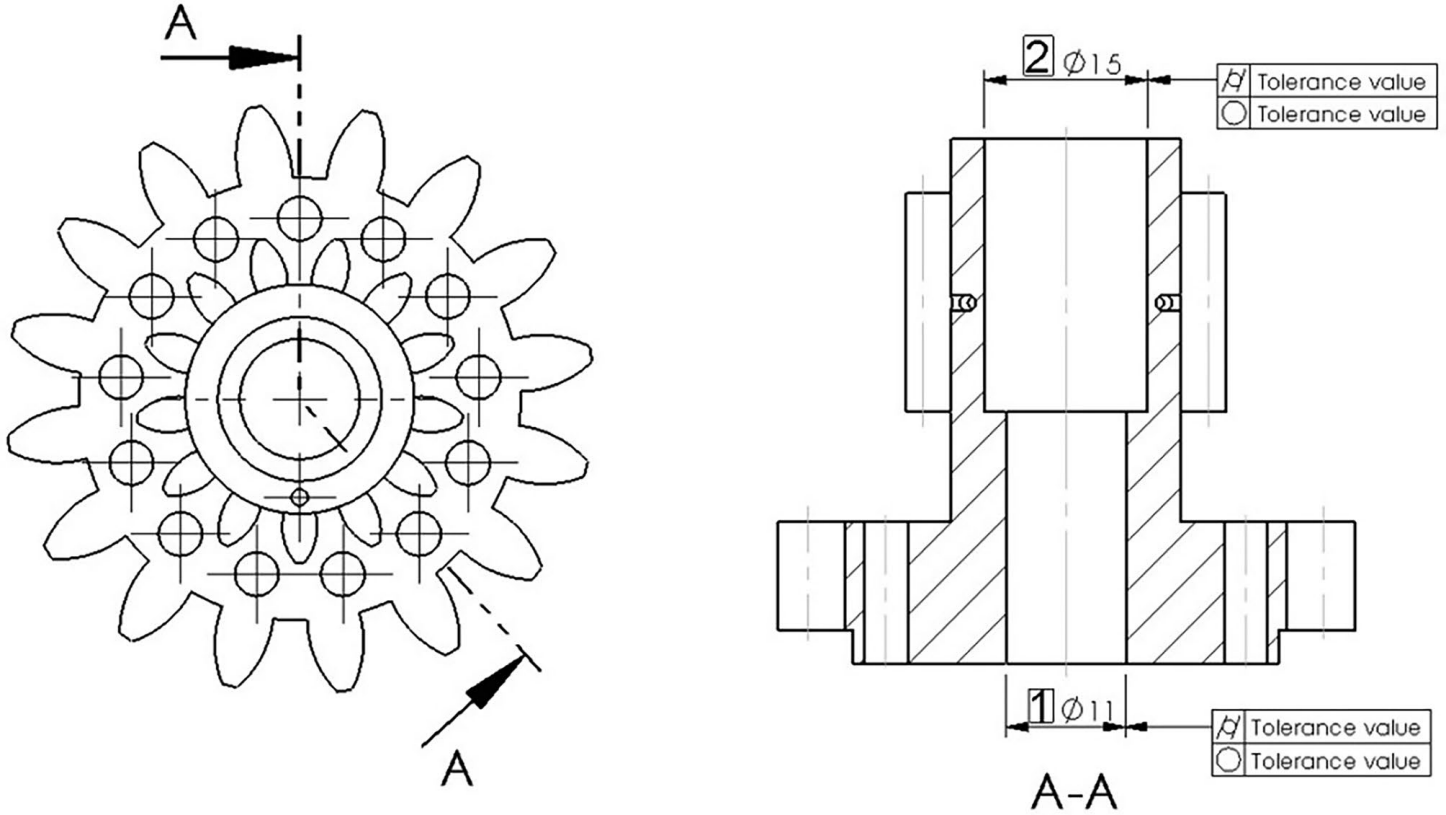
Assessed dimensions and geometrical tolerances

To assess the geometrical accuracy of the manufactured parts, the dimensions and geometrical tolerances shown in Fig. 2 were selected.

In the case of form tolerances, these are tolerances of roundness and cylindricity.

To analyze the effect of model orientation and layer thickness on the accuracy of holes 1 and 2, measurements of the roundness deviations were carried out in five sections. The distance between the sections was 4 mm.

Based on the obtained measurement results, the following parameters were determined:

- roundness deviation RONt, where the associated feature was the least squares circle LSC and the profile was filtered with the use of the Gaussian filter 1–15 UPR,
- the numbers and values of the amplitudes of the three dominant harmonic components of the profiles, and.
- the coefficients of the irregularity of the profile k_1_, k_2_, k_3_ [33] according to the equation.
  1 $${k}_{1}=\frac{{C}_{n2}}{{C}_{n1}};\;{k}_{2}=\frac{{C}_{n3}}{{C}_{n1}};\;{k}_{3}=\frac{{C}_{n3}}{{C}_{n2}}$$
  where RONt is the total roundness deviation of the profile (in fact, it is the distance between the highest peak and the lowest valley of the roundness profile). *C*_*n1*_, *C*_*n2*_, and *C*_*n3*_ are amplitudes of dominant harmonic components of the roundness profile. CYLt is the total cylindricity deviation, where the associated feature was the cylinder calculated by the least squares method LSCY and the profiles were filtered by the Gaussian filter 1–15 UPR. CYLtt is the cylinder taper.

On the basis of obtained values of parameters, a comprehensive analysis has been conducted that consisted of.

- an analysis of roundness measurements results,
- an analysis of irregularity coefficients,
- combined analysis of the joint effect of out-of-roundness, irregularity coefficients, and harmonic components,
- an analysis of cylindricity measurements results,
- an analysis of diameter measurements results, and
- an analysis of surface topography measurements.

### The analysis of roundness measurement results

**Fig. 3:**
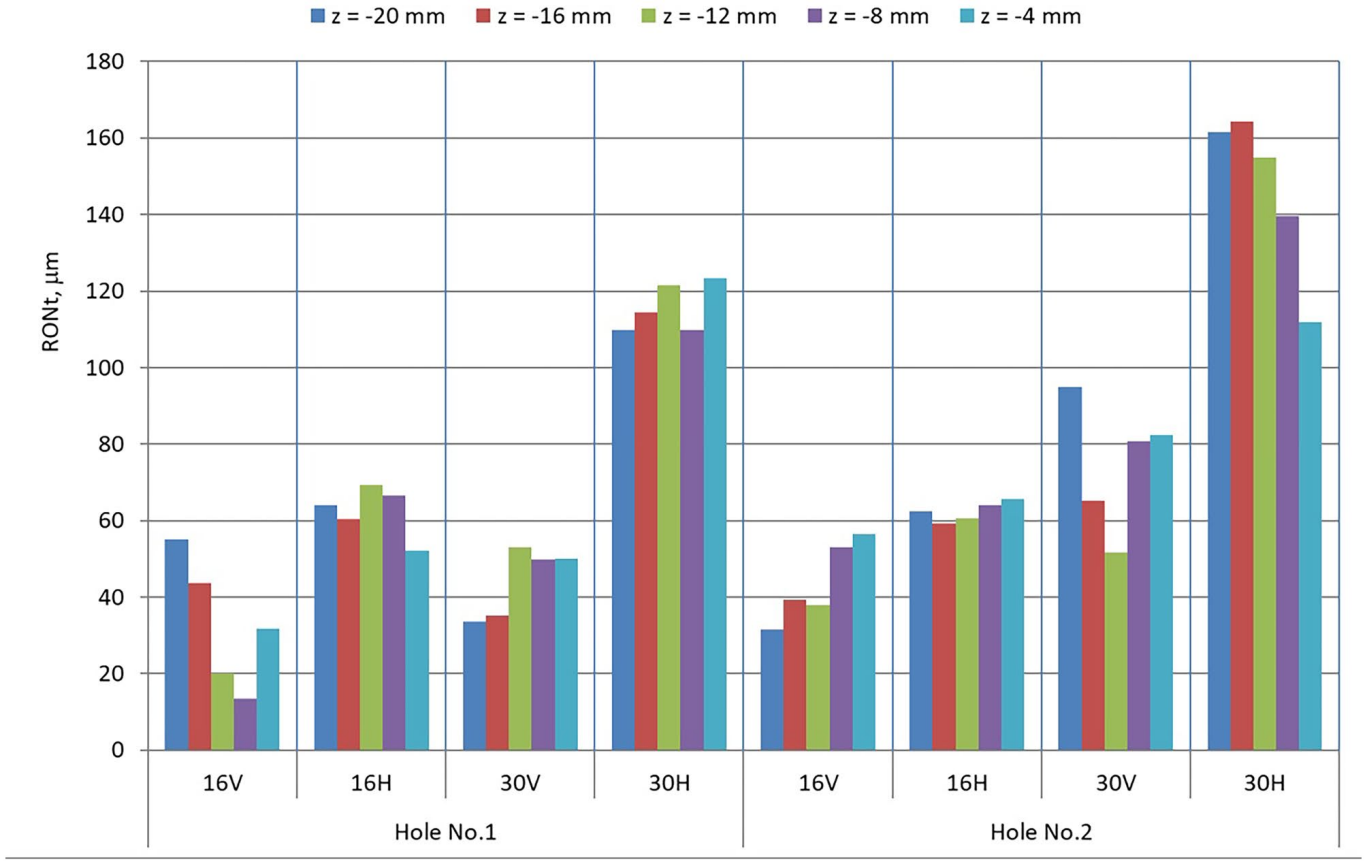
Results of measurements of the roundness deviation RONt of holes 1 and 2

The results of the measurements of the RONt roundness deviation for the printed features, holes 1 and 2 in five cross-sections, are shown in Fig. 3. Parameter *z* defines the distance between the measured cross-section from the face of the sample.

The analysis of the diagram shown in Fig. 3 clearly indicates that samples manufactured in the vertical orientation are characterized by a lower value of roundness deviation. It is also easy to notice that layer thickness affects values of roundness deviation. For most of the samples, we can observe that the lower the thickness layer, the lower value of roundness deviation. Diagrams in Fig. 3 also show that for the horizontal printing, we obtain more uniform values of roundness deviation. For example, for the sample 16H, the difference between minimum and maximum value of the roundness deviation is about 22%, whereas, for the sample 16 V, the maximum difference between the values is about 266%. For other samples, the differences are not so huge, but in all cases, the results for the horizontal orientation are more uniform.

**Fig. 4:**
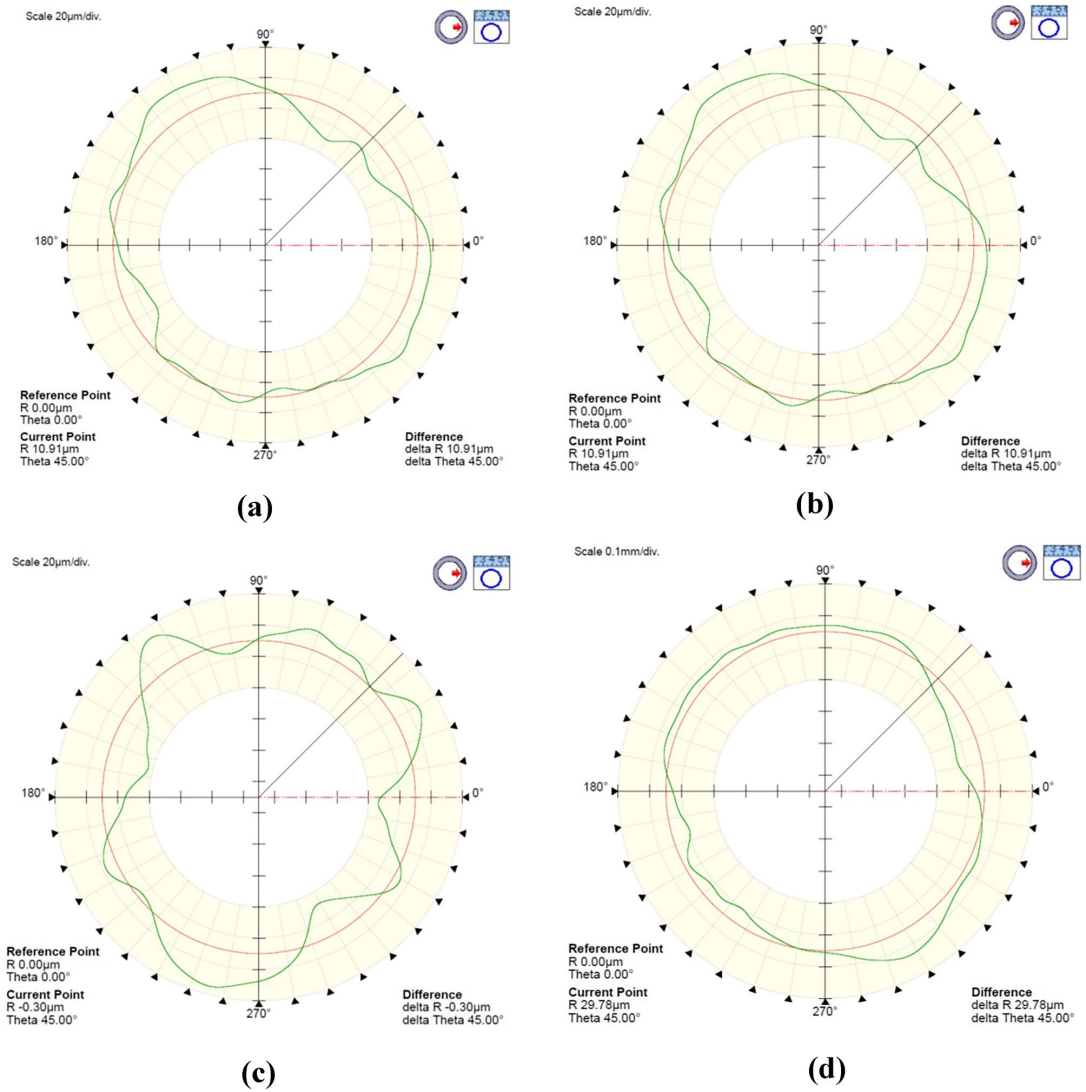
Roundness profiles for the LSC-associated feature applying Gaussian filter 1–15 UPR for the hole no. 2: **a** 16 V, **b** 16H, **c** 30 V, and **d** 30H

Examples of roundness profiles for the associated circle determined by the least squares method and using the Gaussian filter 1–15 UPR for hole 2 are shown in Fig. 4.

An analysis of diagrams for a thickness layer of 16 microns shows that the surface profiles for objects in the horizontal and vertical orientation are very similar.

In turn, for a layer thickness of 30 microns, it is noticeable that the surface profiles of parts manufactured in the horizontal orientation are characterized by smaller local irregularities.

**Fig. 5:**
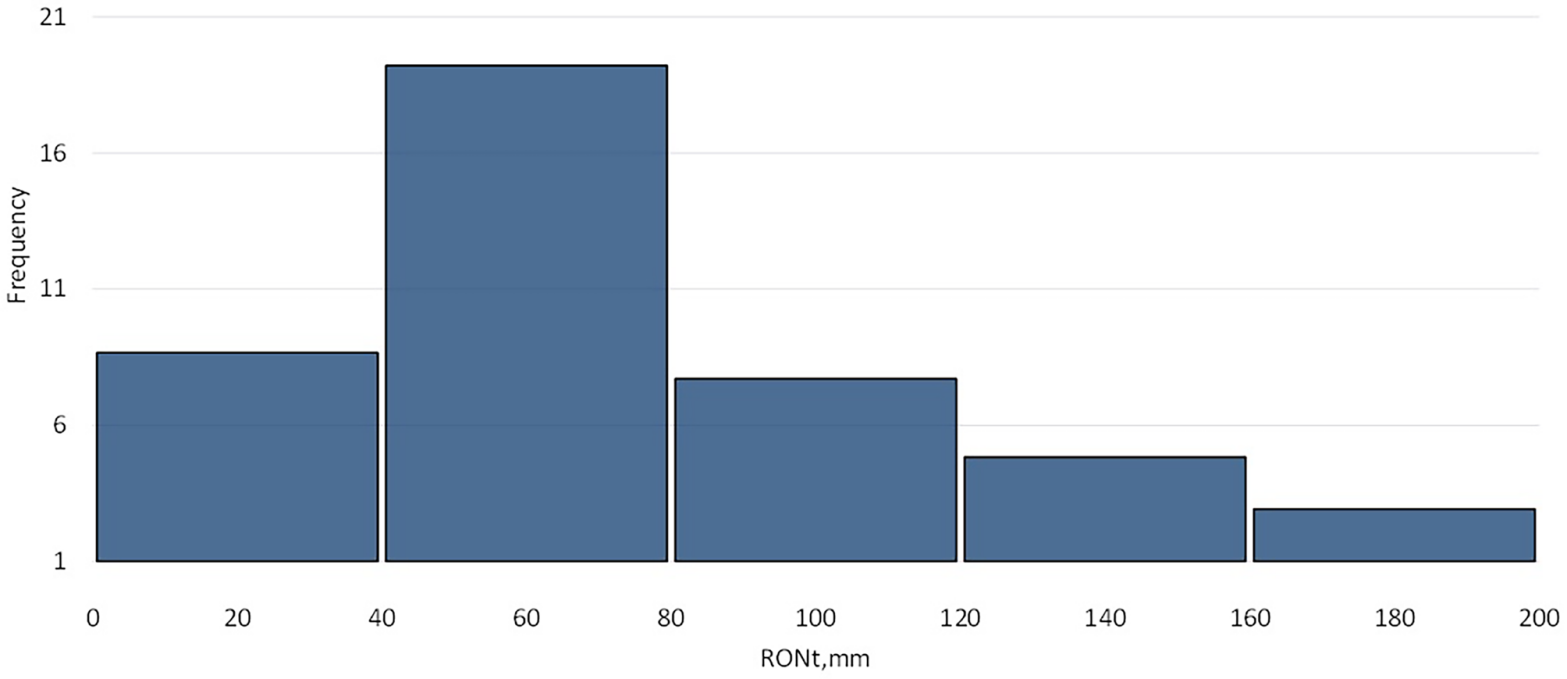
The histogram of the obtained values of roundness deviations RONt

All obtained values of RONt roundness deviations are shown in the form of a histogram in Fig. 5.

The largest number of results of the roundness deviation RONt was obtained in the range from 40 to 80 microns (19 results out of 40 or 47.5%).

**Fig. 6:**
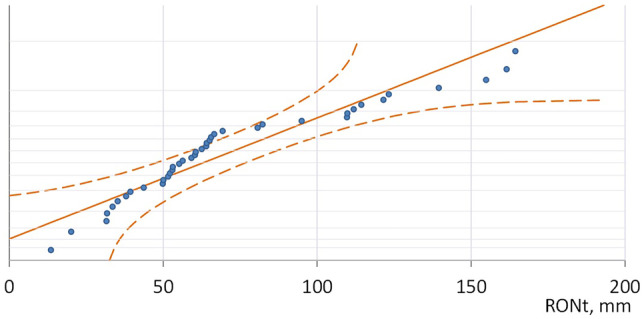
Results of the normality test of the distribution of the results of measurements of the roundness deviation RONt

The Shapiro-Wolf distribution normality test showed that the results obtained do not conform to the normal distribution (Fig. 6).

A test of the average values of roundness deviations for holes 1 and 2 was carried out. There is not enough evidence to conclude that the obtained mean values of RONt roundness deviations for individual holes are different.

The mean value of the roundness deviation for hole 1 with a diameter of 11 mm is slightly lower than the value of the mean roundness deviation for hole 2 with a diameter of 15 mm.

### Irregularity coefficients

The value of the roundness deviation and the profile irregularity are significantly determined by the three dominant harmonic components. In the case when the values of the irregularity coefficients *k*_1_, *k*_2_, and *k*_3_, of the profile are close to one, then it means that it is a complex profile, devoid of one dominant harmonic component.

**Fig. 7:**
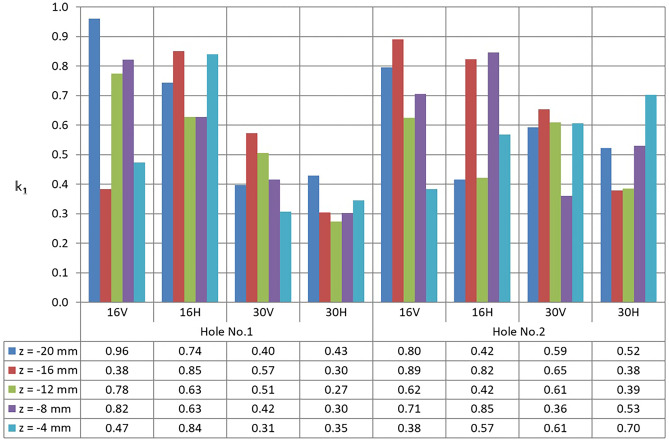
Values of the irregularity coefficient for holes 1 and 2

Figure 7 shows the results obtained for the irregularity coefficient *k*_1_. Parameter *z* defines the distance between the measured cross-section from the face of the sample.

Although the results presented graphically in Fig. 7 are very diverse, some tendencies are noticeable.

For example, one can note that, for a layer thickness of 30 microns, smaller roundness deviation values are achieved than for a layer thickness of 16 microns.

It can be seen that the values of the roundness deviation in individual cross-sections (*z* value) are very diverse and there are no indications that there are areas where smaller values of roundness deviation can be expected.

### The multi-parametric assessment method

To make a comprehensive assessment of the accuracy of the holes in the printed parts, a novel assessment method called a multi-parametric one was used. The new method employs three factors: the roundness deviation RONt, the number of the dominant harmonic component, and the coefficient of the irregularity of the profile *k*_1_. Grades from 2 to 5 were attributed to each factor, then.

The overall rating was a weighted average with the following weights: 0.7 for the roundness deviation, 0.2 for the dominant harmonic number, and 0.1 for the profile irregularity coefficient *k*_1_.

In order to assign appropriate grades to individual results, percentiles were calculated for the set of obtained values of roundness deviations. On the basis of the percentiles, the set was divided into four subsets.

A similar procedure was applied in the case of the coefficient of irregularity of the profile *k*_1_. A summary of the evaluation criteria for each component is presented in Table 3.

**Table 3:** The summary of the evaluation criteria for each component

Grade	RONt, µm	*k*_1_	*n*
5	RONt < 50	k_1_ > 0,74	≥ 5
4	50 ≤ RONt < 63	0,74 ≥ k_1_ > 0,57	4
3	63 ≤ RONt < 95	0,57 ≥ k_1_ > 0,40	3
2	RONt ≥ 95	0,40 ≥ k_1_	2

**Fig. 8:**
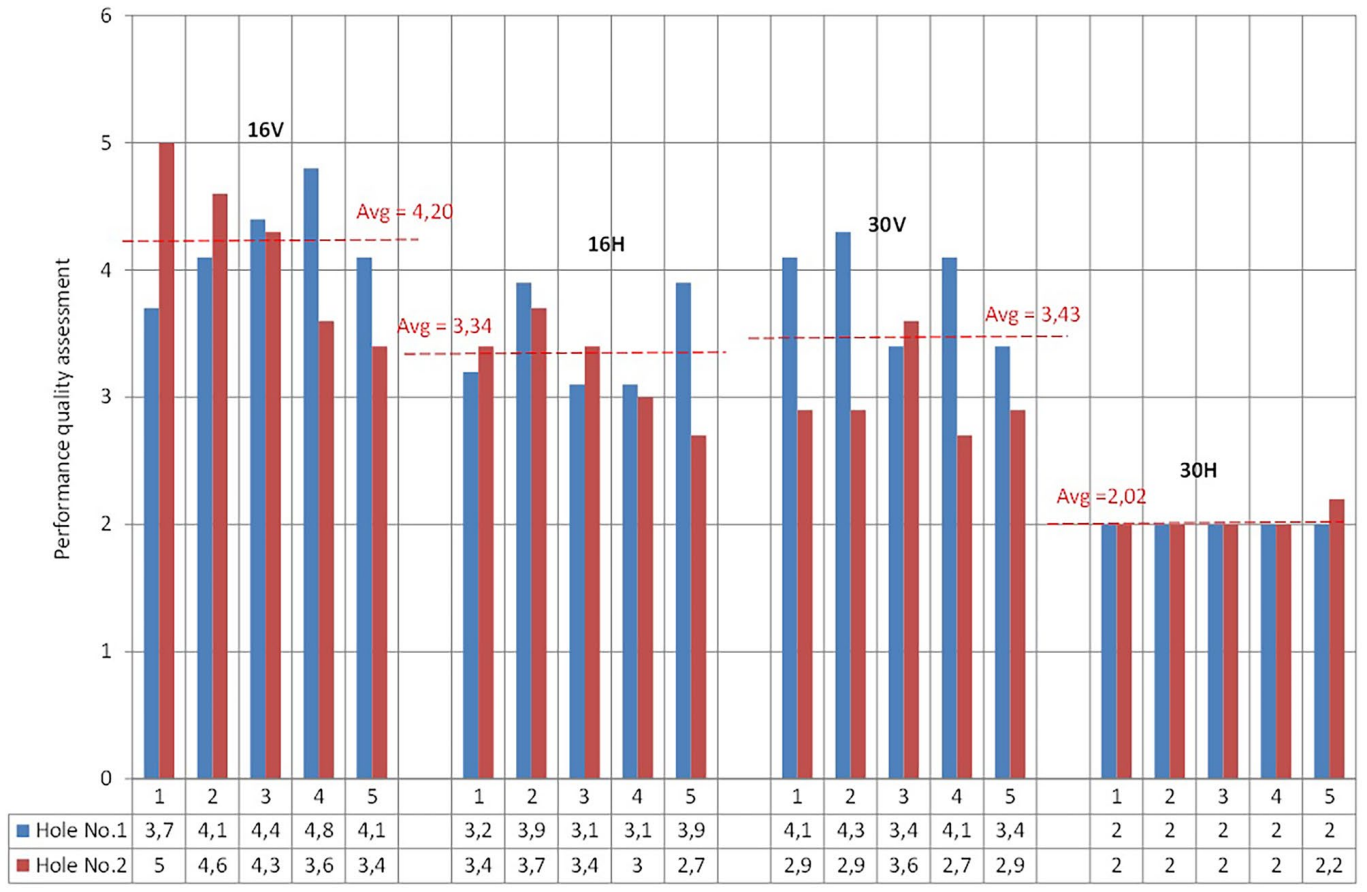
Results of the assessment of the accuracy of the manufacturing of the holes

Using such an adopted scale of grades and weights, an assessment of the quality of the holes was carried out. The results of the analysis are shown in Fig. 8.

The average values of the ratings for each printed element are marked with a red dashed line. The analysis of the results shows that the highest accuracy is characterized by holes made in the 16 V element. The accuracy of the holes in the 16H and 30 V parts is at a similar level. The lowest accuracy was obtained for the holes in the 30H part.

### Results of cylindricity measurements

Measured cylindricity deviations CYLt for the associated feature calculated by the least squares method and filtered by the Gaussian filter 1–15 UPR and the cylinder taper CYLtt have been given in Table 4.

**Table 4:** Results of measurements of cylindricity deviations and the cylinder taper

Notation	Hole no. 1		Hole no. 2	
	CYLt, µm	CYLt, µm	CYLt, µm	CYLt, µm
16V	83,58	− 66,41	88,3	− 94,99
16H	81,52	49,79	101,36	90,90
30V	111,09	− 133,99	109,11	− 73,85
30H	136,60	66,52	185,81	153,1

Negative values of the cylinder taper CYLtt indicate the convergence of the profiles upwards. Based on the obtained results, it should be concluded that there is a correlation between the direction of convergence of the cylindrical profiles and the orientation of the model printout. The holes for the vertical orientation of the print are characterized by negative values of the deviation of the parallelism of the generatrixes of the cylinder – The convergence of the profiles upwards is observed, then. The holes for the horizontal orientation of the print are characterized by positive values of the deviation of the parallelism of generatrixes of the cylinder – The convergence of the profiles downwards can be observed, then.

## Conclusions

The research results presented in the article allow us to draw a number of conclusions regarding the impact of model orientation on the platform and thickness on the roundness and cylindricity deviations of manufactured parts.

The analysis of the RONt roundness deviation value depending on the orientation of the model showed that smaller deviation values were obtained for the vertical orientation of the model. It is noteworthy, however, that in the case of the vertical orientation, the results for individual samples were more diverse than for the horizontal orientation.

The analysis of the distribution of the results of the roundness deviation showed that for the assumed manufacturing parameters, almost 50% (exactly 47.5%) of the obtained roundness deviation values are in the range of 40–80 microns. A more thorough study of the distribution of results (the Shapiro-Wolf normality test) indicated that the distribution of results differs from the normal distribution.

Another aim of the study was also the comparison of the mean values of the roundness deviation values for holes 1 and 2. The analysis of the average values of the roundness deviation of both holes showed that a smaller deviation value was obtained for hole 2. It should be noted, however, that this difference was not significant. For the hole 2, more varied results were obtained than for hole 1. In order to investigate whether the difference between the roundness deviation values obtained for holes 1 and 2 is statistically significant, a test of average values was carried out. The result of this test showed that at the significance level of 0.05, the difference between the obtained values is statistically negligible.

According to the authors, a very interesting tool for comparing the results is the use of a so-called multi-parametric analysis, in which individual parameters are assigned weight coefficients. In the presented article, such an analysis was carried out by taking into account the value of the roundness deviation, the number of the dominant harmonic component, and the so-called irregularity coefficient (see “Sect. 3.3”). Owing to this analysis, we can conclude that the highest accuracy is obtained for holes manufactured in the vertical orientation when the thickness layer of 16 microns has been applied. The accuracy of the holes in the 16H and 30 V parts is at a similar level. The lowest accuracy was obtained for the holes in the part manufactured in the horizontal orientation when the thickness layer was 30 microns.

An analysis of the value of the cylindrical deviation CYLt showed that there is a correlation between the direction of convergence of the cylindrical profiles and the orientation of the model printout. The holes for the vertical orientation of the print are characterized by negative values of the deviation of the parallelism of the generatrixes of the cylinder. The holes for the horizontal orientation of the print are characterized by positive values of the deviation of the parallelism of generatrixes of the cylinder.

Further research activities will be aimed at assessing the impact of the above-mentioned factors on the values of deviations of form, position, and direction for other methods of additive manufacturing. The authors are going to apply coordinate measuring machines and optical systems in their research. In addition, the authors are also going to use computed tomography to evaluate the accuracy of inner dimensions and to observe defects inside the printed part.

## Data Availability

All data are stored at the Laboratory of Computer-Aided Measurements of Geometrical Quantities (Kielce University of Metrology). Data can be provided to the reader at their request.

## Code availability

Measurement data are stored as a part of Calypso software (IGES/DXF files).
